# Methodological Considerations in the Evaluation of RLX‐201 for Skin Health

**DOI:** 10.1111/jocd.71282

**Published:** 2026-11-05

**Authors:** Ume Aiman, Muhammad Salar, Komal Umar

**Affiliations:** ^1^ Liaquat University of Medical and Health Science Jamshoro Jamshoro Sindh Pakistan; ^2^ Chandka Medical College Larkana Sindh Pakistan; ^3^ Peoples University of Medical and Health Sciences for Women Nwb Sindh Pakistan

To the Editor,

We read with interest the report by Draelos et al. [1] which assessed topical RLX‐201 in photoaged skin. The combination of clinical grading, instrumental measurements, histology, and mechanistic experiments is of interest to cosmetic dermatology. Some points of reporting and interpretation, however, should be clarified to aid the reader in interpreting the results and in evaluating the reliability and clinical significance of the results. Firstly, there is not a consistent report of the allocation of participants. The Methods section indicates that 31 participants were randomly assigned to RLX‐201 and 16 to vehicle, but Table 1 in the original study shows 30 and 17, respectively. The group‐specific denominators are not reconcilable between both distributions but sum to 47 participants. The interpretation of a randomized trial [2] is based at the heart of transparent participant accounting. Secondly, the efficacy‐analysis population is not clear. In the Methods section, available data that were gathered prior to the end of the study were retained, while in the Results section, efficacy analyses were performed among those who finished the study. The authors should clarify that with three participant's dropout and not available for follow‐up, the results from their previous observations might also be used to the analyses of efficacy of the study and how missing outcomes were dealt with. This distinction is important since the exclusion of participants or management of missing data without a specific explanation may influence the interpretation of the results from a randomized trial [2, 3]. Thirdly, the statistical reporting would also be improved if it were more specific. In the Methods section Student's *t*‐test for continuous variables and Wilcoxon signed rank test for ordinal grading changes are identified, but in the Results section differences between treatment groups are described. The authors should report which test was employed for each comparison between the different groups and differentiate between within‐group change and between‐group change (vs. the vehicle). Reporting effect estimates for the groups as well as the pre‐specified primary comparison with confidence intervals would help to facilitate interpretation of the magnitude and precision of findings. The authors should also explain whether multiplicity was dealt with [4] with regard to the multiple outcomes and assessment visits. In fourth, the interpretation of histology should be kept proportional with the methods described. Histological assessments are described as exploratory, and staining‐based examination described, with an association of these observations with increased collagen and elastin synthesis and extracellular‐matrix reinforcement. Qualitative staining can show tissue appearance but not prove the extent or magnitude of structural change or increased synthesis unless confirmed by quantitative image analysis or direct measures of synthesis. Quantitative analysis or more conservative language would better match up the interpretation with the evidence [5]. Last but by no means least, two of the authors are associated with Rapalogix Health Inc. Therefore, we urge the clarification of the role of the company, if any, in the design of studies, analysis of data, preparation of manuscripts, and manuscript decision to publish. This is a request for transparency in the evaluation of the study, especially in view of the statement in the data‐availability that there may be commercial restrictions.

Overall, RLX‐201 is an intriguing investigational approach, the clarity of which will likely be enhanced through reconciliation of participant self‐reports, delineation of the analysis population and statistical comparisons, and calibration of histological claims.

## Conflicts of Interest

The authors declare no conflicts of interest.

## Data Availability

Data sharing not applicable to this article as no datasets were generated or analysed during the current study.

## References

[1] Z. D. Draelos , R. C. Mehta , M. Jha , D. Day , and M. K. Randhawa , “A Novel torc1 Inhibitor, rlx −201, Improves Skin Health, Biomechanics, and Extracellular Matrix Integrity in a Double‐Blind Clinical Study,” Journal of Cosmetic Dermatology 25, no. 10 (2026): e71219, 10.1111/jocd.71219.

[2] S. Hopewell , A. W. Chan , G. S. Collins , et al., “CONSORT 2025 Statement: Updated Guideline for Reporting Randomized Trials,” Journal of the American Medical Association 333, no. 22 (2025): 1998, 10.1001/jama.2025.4347. 40228499

[3] I. R. White , N. J. Horton , J. Carpenter , R. I. M. A. S. Statistics , and S. J. Pocock , “Strategy for Intention to Treat Analysis in Randomised Trials With Missing Outcome Data,” BMJ 342, no. 1 (2011): d40, 10.1136/bmj.d40. PMC3230114 21300711

[4] A. J. Vickers and D. G. Altman , “Analysing Controlled Trials With Baseline and Follow Up Measurements,” BMJ 323, no. 7321 (2001): 1123–1124, 10.1136/bmj.323.7321.1123. PMC1121605 11701584

[5] J. E. Sanders , B. S. Goldstein , D. F. Leotta , and K. A. Richards , “Image Processing Techniques for Quantitative Analysis of Skin Structures,” Computer Methods and Programs in Biomedicine 59, no. 3 (1999): 167–180, 10.1016/S0169-2607(99)00003-6. 10386766

